# The Development of Expanded Snack Product Made from Pumpkin Flour-Corn Grits: Effect of Extrusion Conditions and Formulations on Physical Characteristics and Microstructure

**DOI:** 10.3390/foods2020160

**Published:** 2013-05-14

**Authors:** Norfezah Md Nor, Alistair Carr, Allan Hardacre, Charles S. Brennan

**Affiliations:** 1 Foodservice Department, Faculty of Hotel and Tourism Management, Universiti Teknologi MARA (UiTM), Pulau Pinang, 13500 Permatang Pauh, Penang, Malaysia; 2 Institute of Food, Nutrition and Human Health, College of Sciences, Massey University, Private Bag 11222, Palmerston North 4442, New Zealand; E-Mails: A.J.Carr@massey.ac.nz (A.C.); A.Hardacre@massey.ac.nc (A.H.); 3 Department of Wine, Food and Molecular Biosciences, P.O. Box 84, Lincoln University, Lincoln 7647, Christchurch, New Zealand; E-Mail: Charles.brennan@lincoln.ac.nz

**Keywords:** pumpkin, extrusion, screw speed, feed rate, physical characteristic

## Abstract

Pumpkin products confer natural sweetness, desirable flavours and β-carotene, a vitamin A precursor when added as ingredients to extruded snacks. Therefore, a potential use for dried pumpkin flour is as an ingredient in ready-to-eat (RTE) snack foods. Growth in this market has driven food manufacturers to produce a variety of new high value snack foods incorporating diverse ingredients to enhance the appearance and nutritional properties of these foods. Ready-to-eat snacks were made by extruding corn grits with 5%, 10%, 15% and 20% of pumpkin flour. Snacks made from 100% corn grits were used as control products for this work. The effect of formulation and screw speeds of 250 rpm and 350 rpm on torque and specific mechanical energy (SME, kWh/kg), physical characteristics (expansion ratio, bulk density, true density and hardness) and the microstructure of the snacks were studied. Increasing the screw speed resulted in a decrease of torque for all formulations. When pumpkin flour was added the specific mechanical energy (SME) decreased by approximately 45%. Increasing the percentage of pumpkin flour at the higher screw speed resulted in a harder texture for the extruded products. X-ray tomography of pumpkin flour-corn grit snacks showed that increased levels of pumpkin flour decreased both the bubble area and bubble size. However, no significant differences (*p* > 0.05) in bubble wall thickness were measured. By understanding the conditions during extrusion, desirable nutritional characteristics can be incorporated while maximizing expansion to make a product with low bulk density, a fine bubble structure and acceptable organoleptic properties.

## 1. Introduction

Consumer interest in ready-to-eat (RTE) snack foods is growing due to their convenience, value, attractive appearance, taste and texture [1]. Cereal-based extruded snacks are the most commonly consumed snacks [2]. Extruders can blend the diverse ingredients used to develop novel snack foods. The quality of the final product depends on the processing conditions used during extrusion and this includes the composition of the raw materials, feed moisture, barrel temperature, screw speed and screw configuration [3]. Moreover ingredients and formulation play an important role in developing the texture of the extruded product and ultimately the acceptability of the extruded product to the consumer. Additionally, the conditions under which the product is processed will determine the survivability of labile functional ingredients including vitamins, pigments such as β-carotene and volatile flavour compounds. Therefore, processing must be carefully controlled to optimize the survival of functional components.

Most snack foods contain a high proportion of corn, wheat and rice, with potato and oat and other grain products commonly added in smaller quantities. Vegetable powders are occasionally added but are not common in RTE snacks. Pumpkin is a good source of carotenes, which are responsible for the yellow colour of the flesh. It also contains appreciable levels of sugar and pectin. Sugar sweetens products that include pumpkin, while pectin is a soluble fibre that delivers many good health benefits. However, the utilization of pumpkin has been limited to the consumption of fresh products as a vegetable, or thickeners in soups and purees [4] and the dry powders of pumpkin that are also used to adding colour, flavour and thickness in a range of foods including soups and purees. A potential avenue of development for pumpkin based products is for the expanded snack market where pumpkin can be used to naturally enhance the flavour and colour of these products. The incorporation of pumpkin flour that naturally contains 37% sugar and 14% pectin [5] into the extruded snack will change the extrusion system, generally making it more difficult to achieve a highly expanded low density snack. However, the limits for the addition of pumpkin powder and detailed quantitative knowledge of its effects on the physical properties of snacks containing pumpkin powder are unknown. In this study the effect of varying the proportion of pumpkin flour and screw speed on the torque and specific mechanical energy (SME) of the extruder and the quality of final extruded product were studied.

## 2. Experimental Section

### 2.1. Materials

Pumpkin flour (supplied by Cedenco Foods Ltd., Gisborne, New Zealand) and Specification 220 corn grits from Corson Grain Ltd., Gisborne, New Zealand were used. The Proximate composition of corn grits and pumpkin flour, according to manufacturer data are presented in Table 1.

**Table 1:** Proximate composition of raw materials (corn grits and pumpkin flour) used for the extrusion.

Proximate composition	Corn grits	Pumpkin flour
Protein (%)	6.00	7.10
Fat (%)	1.50	3.10
Moisture (%)	10.0	1.80
Ash (%)	2.00	5.70
Carbohydrate (%)	80.50	82.30

### 2.2. Extrusion

**Figure 1:**
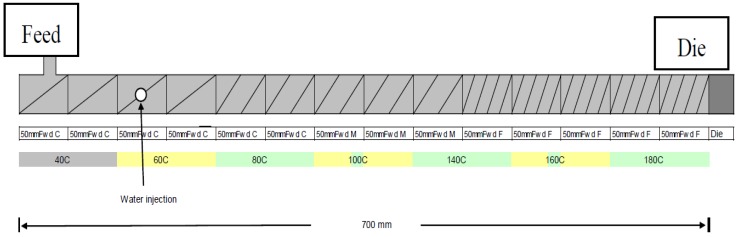
Schematic diagram of screw configuration of the extruder, the angle on the schematic represents screw pitch from the feed at the left hand side to the die on the right hand side.

Expanded snack products were made by extruding finely milled corn endosperm (Specification 220 corn grits) as a control (100%) and corn grits mixed with pumpkin flour added at 5%, 10%, 15% and 20% on a dry weight basis of total mixture 3 kg. These ingredients were added into the extruder at a constant mass flow rate of 12.5 kg/h. Processing was carried out using a twin-screw extruder (Clextral BC21 twin-screw extruder, Clextral, Firminy Cedex, France). The barrel length was 700 mm and the screw diameter was 24.7 mm. The screw configuration (Figure 1) was a four pairs of 13 mm forward pitch screws each 50 mm in length, five pairs of, 10 mm forward pitch screws each 50 mm in length, and five pairs of 13 mm forward pitch each 50 mm in length. The barrel temperatures in each section of the extruder from inlet port to die were set at 40 °C, 60 °C, 80 °C, 100 °C, 140 °C, 160 °C and 180 °C. A single die with a 3.4 mm diameter aperture was used and a knife rotating at 55 rpm cut the emerging product into pellets about 15 m in length. Two screw speeds 250 and 350 rpm were used as treatments for each formulation. 

During this work the processing parameters for the extruder were recorded; these included torque, electrical energy consumption (kW) and from the energy consumption and the total mass through-put, the specific mechanical energy (SME) was calculated as kWh/kg.

## 3. Results and Discussion

In this paper, the effect of adding different proportions of pumpkin flour and operating the extruder at different screw speeds on the associated torque and specific mechanical energy (SME) and the expansion ratio, bulk density, true density, hardness and bubble size distribution of extruded product is reported. 

### 3.1. Extrusion Parameters

**Figure 2:**
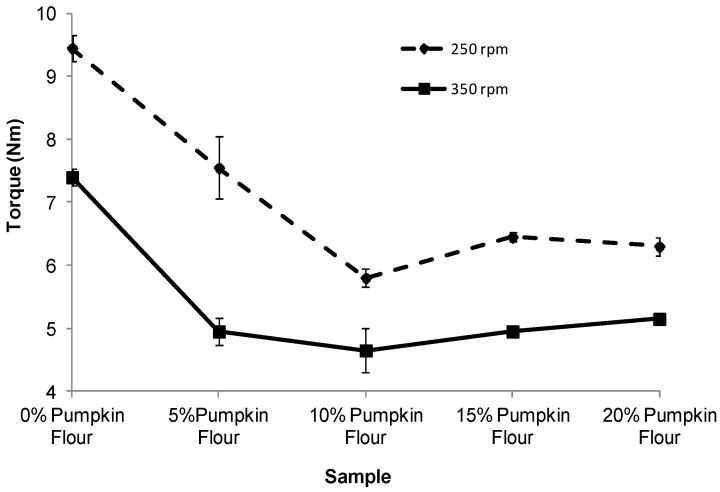
Effect of screw speed and the proportion of pumpkin flour on torque during extrusion.

**Figure 3:**
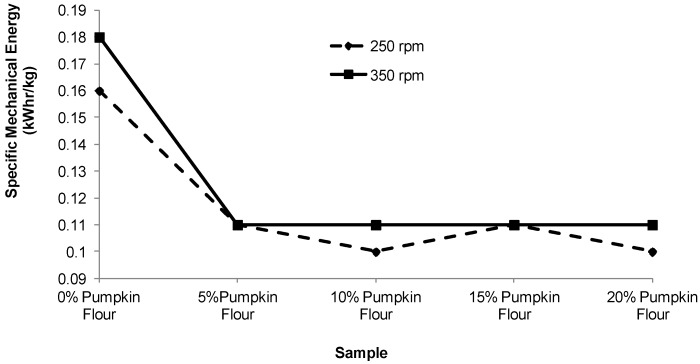
Effect of screw speed and the proportion of pumpkin flour on the specific mechanical energy (SME (kWh/kg)). Each value was an average of duplicate samples.

The torque and specific mechanical energy (SME) response to addition of different proportions of pumpkin flour in the formulation and different screw speeds are presented in Figure 2, Figure 3. Increasing the proportion of pumpkin flour in the formulations decreased torque and SME. However, the rate of reduction in torque and SME was not constant over the range of pumpkin flour added to the formulation. When pumpkin flour was added at 5%, the torque decreased to about 60% of the value for corn grits only and the SME decreased to about 45% of the value for the corn grits alone (Figure 2, Figure 3). At all proportions of pumpkin flour, torque and SME were constant, except for torque at 250 rpm (Figure 2) which was constant at 10% or more of added pumpkin flour. The result also indicated that increasing the screw speed from 250 to 350 rpm at all pumpkin flour proportions decreased the torque during processing but not the SME.

### 3.2. Physical Characteristics

#### 3.2.1. Expansion Ratio and Hardness of Expanded Snack Product

**Figure 4:**
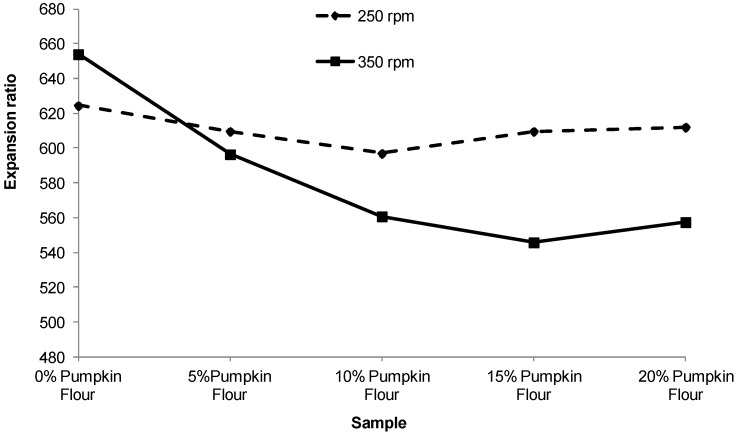
Effect of screw speed and the proportion of pumpkin flour on the expansion ratio. Error bars represent the standard deviation.

**Figure 5:**
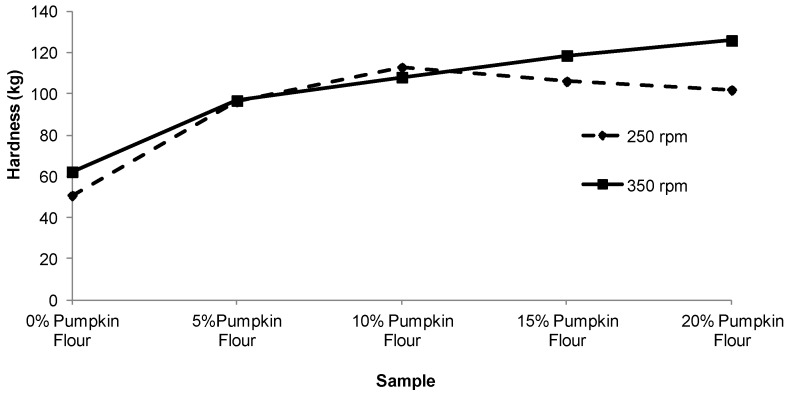
Effect of varying pumpkin flour percentage and screw speed on the hardness of extruded product.

Increasing the proportion of pumpkin flour from 0% to 10% for the 350 rpm treatment decreased the expansion ratio of the extruded pellets by about 15%, but no further change occurred as the proportion of pumpkin flour was increased to 15% and 20%. At a screw speed of 250 rpm the expansion ratio did not change significantly with the proportion of pumpkin flour (Figure 4). As expected, the hardness of the extruded products (Figure 5) varied inversely with respect to expansion ratio with harder products tending to be less expanded although this was less evident for the product extruded at 250 rpm. 

#### 3.2.2. Density of Expanded Snack Product

**Figure 6:**
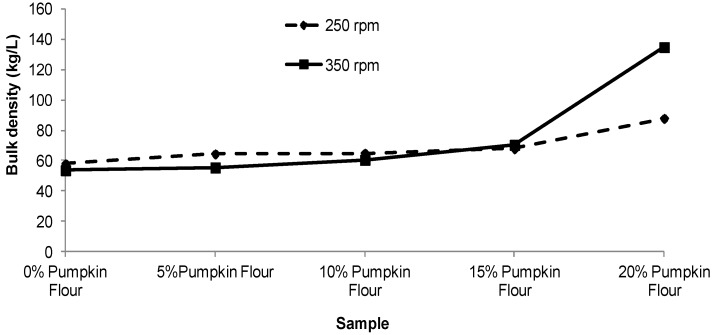
Effect of varying pumpkin flour percentage and screw speed on the bulk density of extruded product. Each value as an average of duplicate samples.

**Figure 7:**
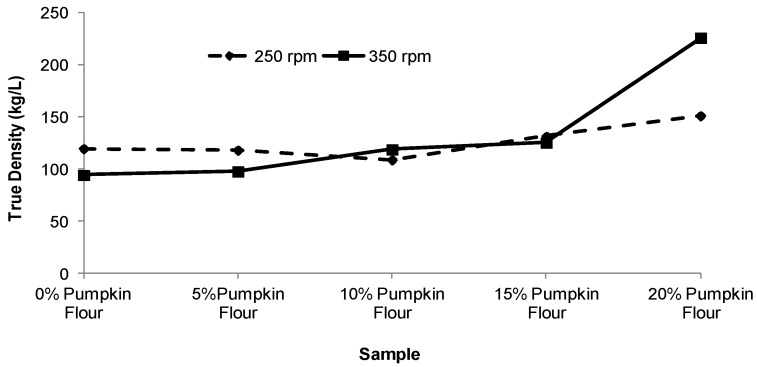
Effect of varying pumpkin flour percentage and screw speed on the true density of extruded product. Each value was an average of duplicate samples.

Bulk density (BD) and true density (TD) of the expanded pellets are shown in Figure 6, Figure 7. The bulk density ranged from 53.7 for the corn pellets to 134.7 kg/L for those containing 20% pumpkin flour. The true density was about 1.7 times greater than bulk density and ranged from 94.4 to 226.0 kg/L. As the level of pumpkin flour added into the blend was increased from 0% to 15% the BD and TD only increased by 5%–15% for the 350 rpm screw speed. However when the level of pumpkin flour was increased from 15% to 20% the BD and TD approximately doubled. At a screw speed of 250 rpm, BD and TD increased by only 25% and 15% respectively as the pumpkin flour content was increased from 15% to 20%.

#### 3.2.3. Microstructure of Expanded Snack Product

**Figure 8:**
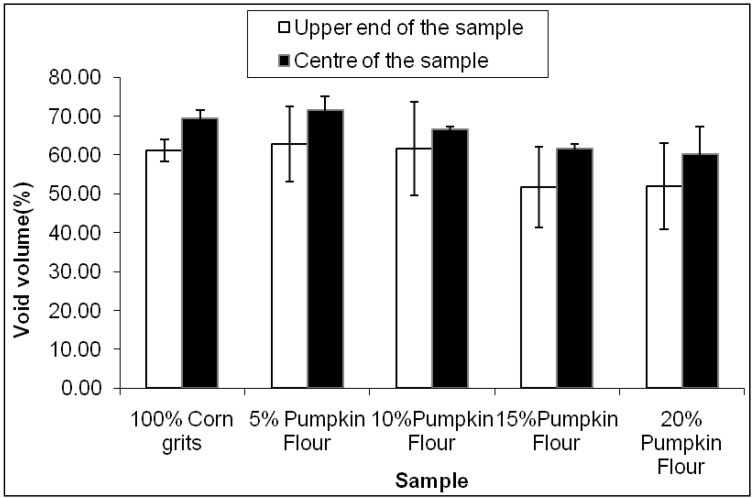
Effect of varying pumpkin flour at constant screw speed (250 rpm) on bubble area distribution at the upper end and centre of the extruded expanded snack.

Due to cost restraints, micro computerised axial tomography (mico-CAT) was only carried out on the extruded product made using the 250 rpm screw speed. The proportion of the cross-sectional area of the extruded pellets that was represented by void area (bubbles) for pellets containing different proportions of pumpkin flour is shown in Figure 8. Near the ends of the pellets, the void area was between 50% and 60% and about 10% less than in the centre of the pellets (Dark shading Figure 8 and Table 2). Towards the centre of the pellets the bubbles increased in size and the void area increased. Increasing the proportion of pumpkin flour although increasing the hardness and density of the pellets, had a relatively small effect on the proportion of void area in the pellets. The mean void area decreased by about 15% for the ends and centre of the pellets as the proportion of pumpkin flour in the ingredients increased. However these differences in void area were not significant, although they were consistent with the significant increases in density and hardness for pellets extruded at 250 rpm.

**Table 2:** X-ray tomography cross sectional of 2-D image (radial): changes of bubble size through out the extruded sample at screw speed 250 rpm (scale 1:2).

Sample	Upper end	Middle
0% Pumpkin flour	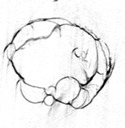	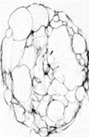
5% Pumpkin flour	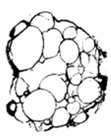	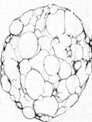
10% Pumpkin flour	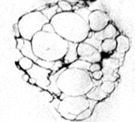	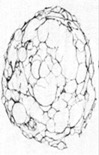
15% Pumpkin flour	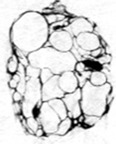	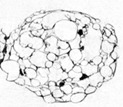
20% Pumpkin flour	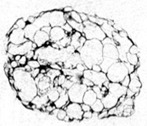	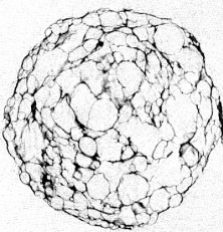

As the proportion of pumpkin flour increased, torque and SME decreased showing that the viscosity of the melt formed within the extruder decreased during processing. This was probably due the high proportion of pectin (14%–22%) and sugar (37%) in the pumpkin flour that reduced the viscosity melt compared with a melt formed from corn grits only. This effect has been previously shown for ingredient mixes that contained high levels of sugars [6]. Melts containing higher sugar levels also decrease the viscosity of the melt as it leaves the die thus delaying setup of the expanding melt. As a consequence, the bubbles of steam cool and the melt collapses to some degree causing shrinkage and decreased the expansion of the extruded product [7].

During this work it was possible to keep the SME near constant for all treatments except the 100% corn grits for which torque and SME were about 64% greater than for the treatments including pumpkin flour. The lubricating effect of the sugar and pectin components in the pumpkin flour were clear although it is surprising to observe the stabilizing effect with addition of 5% of pumpkin flour. This effect is evident throughout this work and the expansion ratio, hardness, densities and probably the void area of extruded product made with 10%, 15% or 20% of pumpkin flour were not significantly different. This may be because the sugar and pectin in the pumpkin flour preferentially coats the working surfaces in the extruder possibly by the formation of a depletion layer. 

As the proportion of pumpkin flour increased from 0% to about 10%, the extruded pellets approximately doubled in hardness but their true density and void areas did not differ significantly. This suggests that the bubble walls in the foamed pellets were stronger and less prone to fracture. Another explanation is that the bubbles increased in size while the walls became thicker; however this is not evident in the micro-CT scan data for the centre region of the pellets.

## 4. Conclusions

This work illustrates that snack foods can be produced from combinations of corn grits and pumpkin products. Although, the texture of this product was about 40% harder than for typical corn based products the bulk density was similar up to the addition of 15% of pumpkin flour. At all levels of added pumpkin powder below 20%, the hardness of the pellets seemed to be determined by the strength of the bubble walls and not by the number and distribution of bubbles measured as the void volume in the extruded pellets or as bulk density.
